# High Speed Quantum Key Distribution Over Optical Fiber Network System^1^

**DOI:** 10.6028/jres.114.010

**Published:** 2009-06-01

**Authors:** Lijun Ma, Alan Mink, Xiao Tang

**Affiliations:** Information Technology Laboratory, National Institute of Standards and Technology, Gaithersburg, MD 20899

**Keywords:** detection time bin shift, fiber network, frequency up-conversion detection, quantum key distribution, BB84, B92

## Abstract

The National Institute of Standards and Technology (NIST) has developed a number of complete fiber-based high-speed quantum key distribution (QKD) systems that includes an 850 nm QKD system for a local area network (LAN), a 1310 nm QKD system for a metropolitan area network (MAN), and a 3-node quantum network controlled by a network manager. This paper discusses the key techniques used to implement these systems, which include polarization recovery, noise reduction, frequency up-conversion detection based on a periodically polled lithium nitrate (PPLN) waveguide, custom high-speed data handling boards and quantum network management. Using our quantum network, a QKD secured video surveillance application has been demonstrated. Our intention is to show the feasibility and sophistication of QKD systems based on current technology.

## 1. Introduction

A quantum key distribution (QKD) system does not transmit secure messages, it creates a shared secret between users over unsecured communication links. The shared secrets are then used to create secure messages that can subsequently be transmitted via conventional IP protocols and channels. QKD systems use quantum states, such as polarization, to encode information on single photons. An initial random key is established by randomly encoding state information on these photons, sending the photons and recovering that state information on the other end of the link. After three additional conventional processing/communication stages, this initial (raw) key is transformed into a secure key. It is not possible to make a perfect copy (clone) of an unknown quantum state [1], thus precise measurement by an eavesdropper is not achievable. The Heisenberg uncertainty principle states that pairs of quantum properties cannot be precisely measured simultaneously; for example, position and momentum. Horizontal-vertical and diagonal polarization of photons are two such pairs.

The idea to use quantum states to securely encode information originated with Stephen Wiesner in 1983 [2], and the idea was taken forward by Charles Bennett and Gilles Brassard in 1984 [3] to develop a QKD protocol. The QKD protocol that they proposed uses four quantum states and is called BB84 (named after the Bennett-Brassard 1984 paper). In 1992, Charles Bennett proposed a simplified version using only two quantum states, thus named B92, to reduce the complexities of a QKD system [4]. These two protocols are the basis of most QKD systems today. The first demonstration of a QKD system was completed in 1989, in which the quantum channel was a 30 cm long path of air in a laboratory. Since then, a number of groups have successfully developed many experimental QKD systems, which were described in a comprehensive 2002 review article [5].

Today’s high-speed QKD technology has become sophisticated, but hasn’t made it out of the laboratory yet. The next step is to integrate these QKD systems into networks and their security protocols using existing communications infrastructure.

A quantum network connects a number of point-to-point QKD systems together so that one can develop shared secrets (secure keys) between users anywhere on the network. A QKD system consists of quantum channels and classical channels. A quantum network would be an embedded sub network within a conventional communication network for the purpose of developing shared secrets, not transporting secure messages. The quantum channels send qbits and the classical channels may be standard IP channels of the conventional network. Networks are commonly divided into three categories, (i) local area network (LAN), (ii) metropolitan area network (MAN) and (iii) wide area network (WAN). The LAN is a short distance network (usually less than 5 km) and can use a star/hub configuration. Low-cost is a primary consideration for a LAN. A MAN is geographically larger than a LAN and usually covers a city area (**~**50 km). A MAN can use a ring or mesh configuration. A WAN (several hundreds km, or even longer), sometimes called a core network or a long-haul network, covers a broad area linking metropolitan areas and crossing national boundaries. A WAN usually uses a mesh network configuration. Longer distances and higher speeds are the main requirements for MANs and WANs.

We have developed several technologies to integrate QKD into such networks. For LANs, our 850 nm QKD system is a good choice, since it uses low-cost Vertical Cavity Surface Emitting Lasers (VCSELs) and Silicon-avalanche photon detectors (Si-APDs). The performance of our 850 nm QKD system exceeds 1 Mbits/s sifted key rate, a common intermediate QKD protocol performance measurement (see Sec. 2 for protocol stages), at 4 km over standard telecom fiber [6, 7]. Further cost reduction is achieved by implementing a detection-time-bin-shift (DTBS) scheme that reduces the number of APDs, with a trade-off of a lower secure key rate [8, 9]. For single photons to traverse the longer distances of MANs, telecom wavelengths (1550 nm or 1310 nm) must be used. We have developed an up-conversion technique based on PPLN waveguides to convert 1310 nm photon into 710 nm photons detectable by low-cost Si-APDs [10, 11]. Using our up-conversion technique, we have attained a performance of about 1 Mbit/s sifted key rate over 10 km of fiber with a low quantum bit error rate (QBER). An entangled photon source at 1310 nm and 895 nm are being developed to achieve greater distances on the 1310 nm side while benefiting from low-cost on the 895 nm side. There is no suitable QKD technology for WANs using a faint laser source, since photons will be attenuated below the noise level over such a long distance and photons can not be copied or amplified. One potential solution for QKD over long distances, other than linking multiple QKD systems together and requiring that the intermediate nodes all be trusted, is a quantum repeater with an entangled-photon-pair source. Currently there are no operational quantum repeaters. To manage high-speed QKD systems, computers alone are not sufficient and dedicated hardware support is necessary. To provide that support we designed and implemented a programmable set of custom high-speed data handling printed circuit boards. These boards allow us to continuously attempt to send photons at GHz rates to retrieve Mbit/s of sifted key, which is a more manageable rate for the remainder of our QKD protocol flow implemented in software [12, 13]. As a first step into quantum networks, we implemented a 3-node quantum network using a Micro-Electro-Mechanical Systems (MEMS) optical switch. We also developed a quantum network manager to coordinate all quantum network activities as well as demultiplex and synchronize the secure bitstream at the top of the QKD protocol flow [14–16]. Using this 3-node network, we developed a video surveillance application secured by our quantum key stream and a one-time pad cipher.

In this paper, we discuss these high-speed QKD systems, presenting their configuration, the concepts that make their operation possible, utilities that automate start-up procedures and operational performance. We also discuss our entry into quantum networking.

## 2. QKD Protocol and its Realization

In this section we will review the BB84 and B92 QKD protocols and discuss the infrastructure that we have designed and implemented to support high-speed QKD and quantum networks.

### 2.1 BB84 and B92 Protocols

The BB84 protocol consists of four stages. The first stage is the transmission of the randomly encoded single photon stream from Alice (the sender) to Bob (the receiver) through an unsecured public link (called the quantum channel) to establish the raw key. This is the most technically challenging stage of the protocol. As mentioned above, horizontal-vertical and diagonal states of photon polarization are a pair of quantum states that cannot be precisely measured simultaneously. The BB84 and B92 protocols use that pair of quantum states to generate random keys. In the BB84 system, each photon is set in one of the four linear polarization states: horizontal-vertical (belonging to the horizontal-vertical basis) or +/− 45 degree diagonal (belonging to the diagonal basis). One of the polarization states in each basis represents a “0” bit value and the other a “1”. Bob randomly chooses to measure each photon in either the horizontal-vertical or diagonal basis. Since there is only a single photon, Bob can only do a single measurement. If Bob chooses correctly, the value he measures will be correct. If he chooses incorrectly, the value he measures will be random. The B92 protocol, a simplified version of the BB84 protocol, uses just two quantum states: one is in the horizontal-vertical basis and the other is in the diagonal basis. Although the B92 protocol is less secure than the BB84 protocol, it is widely used in low cost QKD systems and in laboratory studies. A B92 QKD test-bed could easily be converted to a BB84 system by adding two additional single photon sources and two detectors. In our research, both BB84 and B92 installations are used.

The next three stages of the protocol, common to both BB84 and B92, are conducted over an unsecured public link (called the classical channel, since this can be standard IP communications). These messages must be authenticated and integrity protected to prevent tampering although encryption is not needed since secrecy is unnecessary. The second stage is sifting, where Bob sends a list back to Alice of photons detected and their basis (measurement state), but not their value. Alice retains, from its temporary database, only those entries received from Bob in the correct basis and sends this list back to Bob, who also retains only those entries on this list. For B92, the basis is the same as the bit value, so the basis is not revealed but which photons were received is still used to cull the list. Alice and Bob now have a list of sifted keys. These lists are the same length but may have some errors between them. This QBER is a potential indication of eavesdropping. The third stage is reconciliation to correct these errors. Cascade, and its variants, is the predominant reconciliation algorithm that exchanges parity and error correcting codes to reconcile errors without exposing the key values. This process requires a number of communications between Bob and Alice and results in a list smaller than the sifted list. The fourth stage is privacy amplification, which computes a new (smaller) set of bits from the reconciled set of bits using a hashing algorithm and requires no communication between Alice and Bob. Since the reconciled set of bits was random, the resulting privacy amplified set will also be random. Unless the eavesdropper knows all or most of the original bits, she will not be able to compute the new set.

A conventional threat model assumes an eavesdropper, commonly called Eve, intercepts the photons, measures them and generates new photons based on those measurements, which are sent to Bob. From this attack, Eve will introduce on average a 25 % QBER [3] in the raw key that Bob recovers. Although there are other more complex attacks that involve entanglement, Eve still cannot eavesdrop successfully to obtain the keys without introducing a detectable QBER in the raw key. Furthermore, privacy amplification, the fourth stage of the QKD protocol, can be strengthened to compensate for these attacks when the QBER is within acceptable bounds.

### 2.2 Hardware Support

Single photon transmission is very lossy. Only a few photons in 1,000 get through and that amount drops as the transmission distance increases. NIST’s focus on QKD has been to achieve high-speed secure key generation. To generate secure keys at Mb/s rates requires sending photons at GHz rates, and to operate continuously at GHz rates requires hardware support and a synchronous communication model. We designed and implemented a pair of printed circuits boards (PCBs) for this hardware support. This has greatly improved the overall key generation rate as more of the QKD protocol, originally implemented in software, is moved to hardware.

**Fig. 1:**
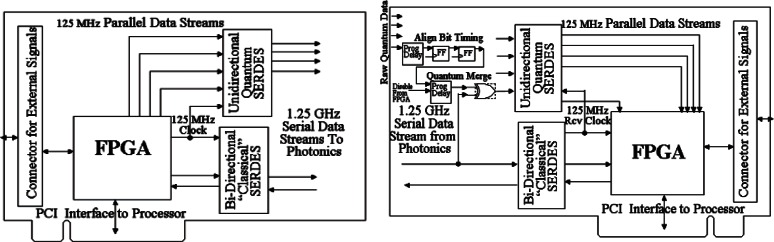
PCB Functional Block Diagrams of Alice (left) and Bob (right).

The major functional portions of the PCB pair are shown in Fig. 1. Each PCB contains a field programmable gate array (FPGA) chip and a pair of serializer/deserializer (SerDes) chips. Each SerDes can support up to four bi-directional Gigabit channels. The FPGA exchanges 10-bit parallel data with the SerDes at 125 MHz. The SerDes converts between 10-bit parallel data at 125 MHz and serial data at 1.25 GHz. For synchronous communication on the classical channel, no additional support is needed. Synchronous communication requires data to be sent continuously so that the receiver can recover the transmit clock and use it to correctly extract the bits of the data stream. When there is no real data to send, predefine idle characters are sent to fill any potential absence of data. Clock recovery is an important aspect of synchronous communication because although the transmitter and receiver use similar clocks (oscillators), the clocks are not exactly the same. If an inexact clock were used to extract bits from the data stream it would result in errors. For example a typical 125 MHz oscillator has a precision of about 10^−5^, which is +/− 1,250 Hz from the rated frequency. This could result in a difference of up to 2,500 clocks per second between the transmitter and receiver.

We treat each random, relatively slow detector signal as a high speed serial data stream on a separate quantum channel. The serial data rate determines the time bin resolution of our detection events. The data in each quantum channel is unsynchronized, sparse and random. A SerDes requires a continuous, synchronized data stream since one of its functions is to recover the clock of the received data stream. To alleviate this problem we developed special circuitry to condition and prepare the quantum data for processing by the SerDes. As the quantum signals arrive at Bob’s PCB, their phase (sub-bit timing) is aligned by a programmable delay chip to a GHz clock driving a pair of flip-flops. That GHz clock is the recovered clock from the received classical channel and is identical to the transmission clock. Because of jitter, the quantum signals are not stable and so we use flip-flops to stabilize them. The stable flip-flop output is re-aligned with the replicated classical channel data stream by a second programmable delay chip and then XORed with the replicated classical serial data stream and sent to the SerDes as a synchronous signal. This XOR process “piggybacks” the sparse quantum data stream onto a well-formed synchronous data stream that can now be processed by the SerDes. Once processed by the SerDes, the parallel data is passed to the FPGA where it is again XORed with the now parallel classical data stream, leaving the original sparse quantum data stream. This dual XOR process to piggyback the sparse quantum stream onto the classical data stream and then remove the classical data is necessary since the FPGA is not fast enough to directly sample signals at GHz rates. Once the sparse quantum stream is inside the FPGA, we can search it in parallel for a “0” to “1” transition that designates a photon detection event. Its bit position is the time bin in which it occurs. Part of the startup configuration procedure involves aligning the quantum channels with the classical channel. This determines sub-bit time settings of the delay chips and a multiple-bit time delay setting within the FPGA.

**Fig. 2:**
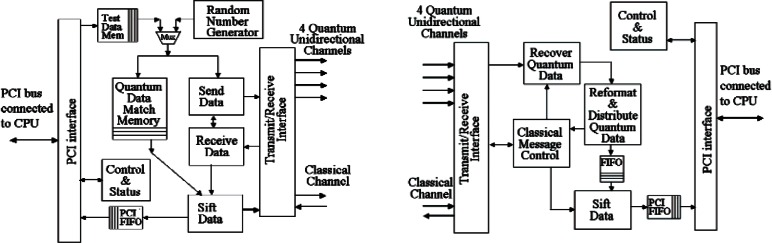
FPGA Functional Modules of Alice (left) and Bob (right).

To explain the operation of the FPGA firmware, we walk through the flow of the modules shown in Fig. 2. The Random Number Generator module on Alice’s FPGA generates two bit-streams of pseudo random data at up to 1.25 Gbit/s each; one stream for the bit value and the other for the basis. Their 2-bit combinations define the four quantum polarization states. These streams are temporarily stored in the Match Memory as well as passed to the Send Data modules where each 2048 bit pairs are grouped into a packet of state information. Each packet is then passed to the Transmit/Receive module where they are synchronously sent to Bob on the quantum channel along with a “Sync” message on the classical channel. These electrical signals are sent from the PCB to the photonics, where they are shaped and converted to optical signals for the classical channel and single photons for the quantum channels. On the destination side of the channels the process is reversed and the recovered optical signals are converted back into electrical signals.

When a “Sync” message is received by the Transmit/Receive module in Bob’s FPGA, it begins the capture of one packet’s worth of data from the Quantum channels. Although the first quantum bit leaves Alice at the same time as the first bit of the “Sync” message, it can arrive sometime later than the “Sync” message since quantum data follows a different detection path than the classical data. If it arrives earlier its an error. We measure this bit delay and specify, via the PCI interface, its value to the FPGA to provide the necessary compensation. Four quantum packets are captured in parallel, one on each quantum detector and each detector has its own delay value. The four quantum packets are passed to the Recover Quantum Data module where they are aligned and then searched for rising edges that denote a detection event. The location within the quantum packet and the associated quantum channel of all detection events (i.e., tagged time bin, basis and value) are passed to the Reformat & Distribute Quantum Data module. This module reformats the data into a set of triples consisting of packet location, basis and bit value. For each packet this set is temporarily stored in a FIFO and also passed to the Classical Message Control module to be sent back to Alice for sifting. The data sent back to Alice does not contain the bit values of this raw key.

Sifting allows Alice to discard bits from its temporary database that were never received by Bob as well as detection events measured by Bob in the wrong basis. When Alice’s Receive Data module gets a packet’s worth of triples, it passes that information to the Sift module. The Sift module compares the basis value of each triple against the correct value stored in the Quantum Data Match Memory. If they match, then the bit value stored in the Match Memory is placed in the PCI FIFO since it is missing from the triple, forming Alice’s stream of ordered Sifted bits. That triple is also sent back to Bob as an acknowledgement. When Bob’s Classical Message Control module gets the acknowledge list, Bob passes that list to its Sift module that compares it against the list in the Temp FIFO and discards all entries that aren’t on the acknowledge list. For those items that are on the list, the bit value is placed in the PCI FIFO forming Bob’s stream of ordered Sifted bits. These Sifted bits are passed to an application program running on the CPU by a device driver in the operating system through a DMA (Direct Memory Access) transfer. DMA is a fast memory transfer that does not require CPU intervention, thus allowing the CPU to continue computation during the transfer.

By using an FPGA in our design we have been able to revise the operation of our system to accommodate and handle nuances with the photonics as well as testing and configuration of our QKD system. The most notable feature has been the Test Data Memory feature that allows us to load any data patterns of quantum bits from an application on Alice’s CPU and send them to Bob in a number of differently controlled packets. Through this feature we are able to test the network and compare observations against known data. We also use this feature to align the quantum channels by sending known data to Bob in order to determine the delay needed for each quantum channel. Other features that have been added include “spacing” in which we can group a number of sequential time bins together that simulates a larger detection window. Along with spacing we can filter out portions of that window to reduce the effects of jitter on the quantum channels. After a photon has been detected we can temporarily disable photon detection for a short time to prevent registering an after-pulse from the detectors, a condition that could introduce additional errors and affect the security of the key. We can reduce the single photon transmission rate to operate with quantum sources and detectors of much lower speeds.

### 2.3 Reconciliation and Privacy Amplification

To match the performance of our hardware the reconciliation and privacy amplification algorithms [17] also had to be enhanced to achieve significant performance improvements. These enhancements include treating distinct data subpopulations differently, adopting forward error correction and using large sequential chucks of data from the sifted data stream. As a result, the numbers of iterations and round trip communications have been reduced. We distinguish three data subpopulations, as corrected, uncorrected and uncorrectable. We divided each large chunk of bits into small segments (5 – 100 bits each) and categorize each segment as belonging to one of these three sub-populations. We consider a segment “uncorrectable” if we detect three or more bits in error. Uncorrectable segments are discarded as they are discovered, thus eliminating expending effort and time to recover a small amount of bits. Forward error correction is much more efficient than parity alone, but care must be taken to use codes that don’t expose too much information about the data being corrected. Large chunks of data allow a significant margin for bits to be discarded to protect any that may be exposed and still retain a significant portion of bits. We use chunk sizes in the range of 64 K to 1 M bits.

The reconciliation algorithm conducts repeated passes on the data until the estimated error rate falls below a preset threshold. In each pass, both Alice and Bob randomly reorder the bits using an identically seeded pseudo-random number generator algorithm, using a new seed each time they reorder. These seeds are extracted from the previous set of privacy amplified keys, except for the first dataset, and are never transmitted, only used internally, thus never exposed to eavesdropping. The reordered set of bits is divided into small segments and the parity, even or odd, for each segment is computed. Alice sends Bob her parity list to compare against his. On the first pass, Bob uses the parity lists to estimate the initial dataset QBER and sends that information back to Alice. When entries in the two parity lists differ, Bob computes a Hamming error correction code on that segment and sends that list back to Alice. No correction codes are computed for segments whose parities match. Alice uses that Hamming code list to identify segments needing correction and attempts a 1-bit correction to segments where feasible, placing those segments into the corrected subpopulation, and when not feasible marks those segments as uncorrectable. Alice makes a list of the processing done on each segment. This list is also sent to Bob. Both Alice and Bob use that same list to determine which segments to keep and which segments to discard. For those segments kept, a number of bits are discarded based on the information exposed from the parity and error correction codes that were exchanged for those segments. Based on the corrections made, the remaining error rate is computed and used to determine if another pass is needed. During each pass, the corrected and uncorrected subpopulations are handled separately. Usually during a number of passes, only one of these subpopulations requires processing, further increasing efficiency. Lists are compiled in their entirety and then sent, thus increasing communication efficiency by reducing overhead. When reconciliation completes, both Alice and Bob compute a 64-bit hash code on the remaining bits. If the hash codes don’t match, the entire chunk is discarded. If they match, the remaining bits are then privacy amplified.

Privacy amplification [18, 19] is a hash function transformation process that further reduces the amount of key in a manner that eliminates whatever information an eavesdropper might know. The amount of information an eavesdropper might know is based on the QBER estimated from the sifted bits determined during reconciliation. The QBER is entirely attributed to potential eavesdropping, rather than distributing it between eavesdropping and system losses. Both Alice and Bob perform identical privacy amplification without exchanging any information. We build a matrix, D, from the reconciled bits of dimension N × 1024, N = reconciled bits/1024. We also build a matrix, G, which consists of randomly generated data of dimension 1024 × S, where S < 1024 and depends on the QBER. The higher the QBER, the smaller the value of S. Both Alice and Bob generate matrix G using the same identically seeded pseudo-random number generator algorithm. From these, we generate a privacy amplified matrix, A, of dimension N × S, whose i-th row (1..S) is obtained by XORing selected rows of D. The i-th column (1..S) of G consists of a 1024 element vector that selects which rows (1..1024) of D are to be XORed together. The computational complexity of this operation is N × N/2, i.e., of order N^2^.

**Fig. 3:**
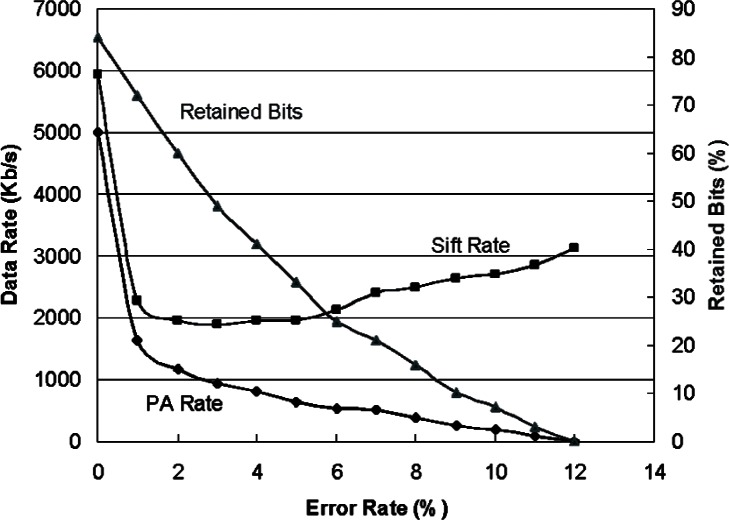
Secure Key Generation Capacities of our Reconciliation and Privacy Amplification Software Implementation.

To increase the performance of these algorithms our implementation uses threads [20] to spawn a number of lightweight parallel tasks. Each parallel task performs the complete reconciliation and privacy amplification algorithms. A separate task accesses and parcels out the sifted bits from the PCB. Another task collects the privacy amplified bits for distribution to security applications and acts as our session key manager. Each reconciliation and privacy amplification task is categorized as coarse grain computation because they require large amounts of computation. Coarse grain computation is known to execute efficiently in a parallel processing environment and in our limited experiments on a few processors we have seen linear speedup as the number of processors increase. The performance of our infrastructure flow is shown in Fig. 3. These measurements are for a dual processor system. Running on a single processor results in half the privacy amplification data rate. The left axis shows the privacy amplified key generation rate and the sifted key rate needed to sustain it as a function of the QBER. The right axis shows the percent of the sifted key that is retained after reconciliation and privacy amplification, as a function of the QBER. As the QBER increases, the privacy amplified key rate and bits retained decrease as expected, but the sifted key rate required to sustain that rate actually increases. This occurs at high QBERs because many of the bits are quickly relegated to the uncorrectable subpopulation and discarded. Also the number of bits left to be privacy amplified, an N^2^ operation, is smaller.

## 3. High Speed QKD Systems for Local Area Network

Our 850 nm QKD system was designed for economical operation over short distances and as a result is a good candidate for use in a LAN. It uses low cost commercial VCSEL and Si-APDs. The single photon attenuation is acceptable over short distances in LAN optical fibers. Si-APDs can operate in free-running mode and their jitter response is only a few hundred ps, which allows our 850 nm QKD system to operate at clock rates over a Gbps. Currently, several groups have implemented 850 nm QKD systems over optical fiber and free-space [21–23].

### 3.1 System Configuration

**Fig. 4:**
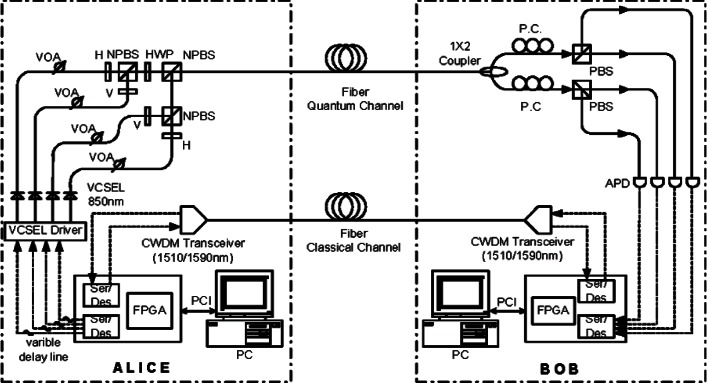
Schematic diagram of our BB84 QKD system; VCSEL: Vertical-Cavity Surface-Emitting Lasers; Pol.: Polarizer; VOA: Variable Optical Attenuator; NPBS, Non-polarizing Beam Splitter; P.C.: Polarization Controller; FPGA: Custom printed circuit board controlled by a field-programmable gate array; PCI: PCI bus; PBS: Polarizing Beam Splitter; Solid line: Optical fiber; Dotted line: electric cable.

Figure 4 shows a schematic diagram of our fiber-based BB84 QKD system, which uses a pair of our PCBs to process the data at a continuous high data rate [13] to create a shared sifted key according to the BB84 protocol. Two 1.25 Gb/s coarse wavelength dividion multiplexing (WDM) transceivers form the bi-directional classical communication channel operating at 1510 nm and 1590 nm. Bob’s PCB recovers Alice’s clock from the classical channel, allowing it to synchronize data with Alice. The precision of this synchronization dictates the resolution of a detection event time bin.

Alice’s PCB generates an 800 ps electrical pulse— full width, half maximum (FWHM) every 1600 ps (625 MHz) on the randomly selected quantum output. Each of the four outputs drives a 10 Gbit/s 850 nm VCSEL that generates a laser pulse. The intensity of the laser pulse is then attenuated by variable optical attenuators (VOA) to the single photon level. A linear polarizer and a half-wave plate (HWP) sets the polarization orientation, −45°, +45°, 0° or 90°, that corresponds to the output path. These four output streams are combined into a single stream by non-polarizing beam-splitters (NPBSs) and then sent to Bob over the quantum channel. The mean photon number (μ) at Alice’s output is set to 0.1, therefore on average, Alice emits one photon every ten pulses.

At Bob, a 1 × 2 non-polarizing single-mode fiber coupler performs a random choice of polarization basis measurement, either the horizontal-vertical basis or the diagonal basis. After the coupler, a polarization compensation module recovers the photon’s polarization state and a polarizing beam-splitter (PBS) separates the photons by their polarization directing them to a Si-APD (Perkin Elmer SPCM-AQR-14) [24] that feeds Bob’s PCB. This process separates the photons into four paths, corresponding to the four BB84 encoding states. A photon measured in the wrong basis would be randomly detected as a “0” or “1”.

Some initial setup is needed before the system starts. Bob cooperates with Alice to perform polarization compensation to recover the photon’s polarization state that may change during transmission through fiber. Since the data paths for quantum and classical channels are different, Alice and Bob need to align the timing between the quantum channels and the classical channels. The polarization compensation and timing alignment are described in the following sections.

### 3.2 Polarization Compensation

A photon’s polarization state as it travels through fiber may randomly change due to strain, vibration or temperature changes on the fiber. For an operational system, it is important to develop a polarization compensation mechanism to automatically trace the drift and recover any polarization transformation otherwise the QBER will increase and the secure key rate will decrease. Auto-compensation can be done either actively [25] or passively [26]. We used the active approach in our QKD system, since the passive method has some critical issues, such as back-scattering, that cannot be solved easily.

**Fig. 5:**
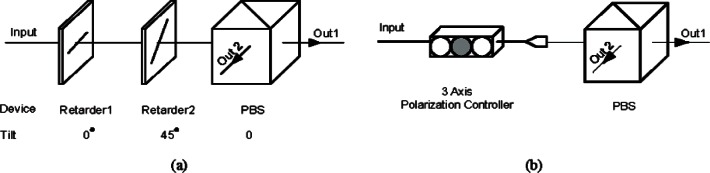
Two kinds of PRAC setting: Liquid Crystal Retardance (a) and Piezo Polarization Controllers (b).

We developed two types of polarization controllers for a polarization recovery and auto-compensation (PRAC) subsystem: one is LC PRAC using liquid crystal retarders (LCR) [27] and the other is PZ PRAC using Piezo Polarization Controllers [28]. Two pairs of LCRs are used in the LC PRAC. Each pair forms a polarization controller for one of two output arms of Bob’s 1 × 2 coupler of Fig. 4. The axes of the LCRs in the pair are pre-aligned with the PBS, as shown in Fig. 5(a). The slow axes of the two LCRs are aligned to the passing axis of PBS by 0° and 45° respectively, while PBS splits the 0°-component of the signal to output port 1 and the 90°-component to output port 2 (+/− 45° for the other basis). The retardance (*α*, *β*) of the LCRs is determined by an applied voltage. In Eq. (1) we show the transformation of the Jones vectors of a PRAC. With the proper applied voltage, we can ideally rotate the received signals to arbitrary polarization states

$$\left\{ \begin{array}{l} E_{out1}=\left[\begin{array}{cc} 1 & 0\\ 0 & 0 \end{array}\right]\;\left[\begin{array}{cc} \cos(\beta /2) & i\sin(\beta /2)\\ i\sin(\beta /2) & \cos(\beta /2) \end{array}\right]\;\left[\begin{array}{cc} e^{i\alpha /2} & 0\\ 0 & e^{i\alpha /2} \end{array}\right]\underset{in}{E}\\ E_{out2}=\left[\begin{array}{cc} 0 & 0\\ 0 & 1 \end{array}\right]\;\left[\begin{array}{cc} \cos(\beta /2) & i\sin(\beta /2)\\ i\sin(\beta /2) & \cos(\beta /2) \end{array}\right]\;\left[\begin{array}{cc} e^{i\alpha /2} & 0\\ 0 & e^{i\alpha /2} \end{array}\right]\underset{in}{E} \end{array}\right..$$ (1)

The PZ PRAC uses two in-line polarization controllers. Each controller consists of three Piezo-driving phase retarders. Each retarder’s axis is independently controlled by a piezo driver. The phase retardance of the three retarders is fixed at π/4, π/2, and π/4 respectively. We can realize arbitrary polarization transformations by setting the axis of each phase retarder. The structure of our PRAC is shown in Fig. 5(b). The LC PRAC has to be aligned with PBS while the PZ PRAC does not since it can achieve an arbitrary transformation. On the other hand, the PZ PRAC is fiber based with virtually no insertion loss, though it may drift slowly. The PZ PRAC response time is faster, 30 μs vs. 100 ms for the LC PRAC. But the PZ PRAC exhibits poor repeatability that causes extra search time. Overall the PZ PRAC is faster.

The extinction ratio (ER) in polarization encoding QKD systems is defined as the ratio of the correct photon counts to the incorrect counts in compatible measurement bases. For example, the ratio between the photon counts of the two output ports of the PBS for a stream of photon of the same basis and bit value. In B92, the ER is the ratio of the counts in compatible detection bases to the counts in incompatible detection bases. For example, the ratio between the counts of the two output ports of the PBS once a photon stream of all the same value is sent. Polarization drift in the transmission fiber induces fluctuation of the ER, which directly influences the QBER. The extinction ratio can be measured by turning on and off corresponding VCSELs and comparing the counts from the Si-APDs. As a result, the ER is a suitable feedback signal for PRAC.

We have developed a program that can measure the Si-ADP counts and compute the ER while it controls the VCSELs, via communication with Alice, and adjusts the polarization controller voltages to achieve PRAC. The procedure has two stages: First a coarse-step search is performed to find the optimal area and then a fine-step search finds the optimal point in that area. This procedure is run at startup and then can be invoked periodically (e.g., every 15 minutes) or whenever the QBER increases. The procedure first checks the ER at the current settings, if the ER is below 20 dB, the procedure will restart. Otherwise, the procedure does a fine-step search of the local area for the highest ER point, if that point is not the current point, change to that point, recompute the new local area and repeat until the point doesn’t change.

**Fig. 6:**
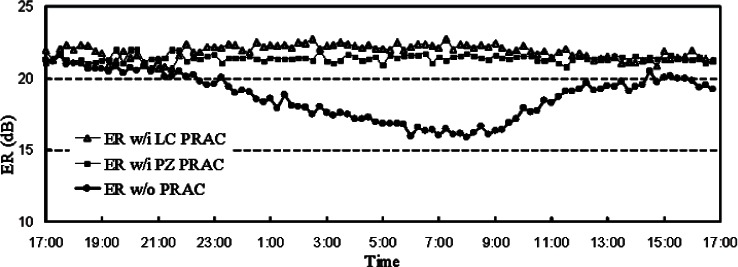
The extinction ratio with/without a PRAC sub-system. With PRAC, the ER is collected immediately after each PRAC tracking operation, which is performed every 15 min over 24 h. Without PRAC, the ER is measured every 15 min but the system is untouched during the whole 24 h.

We studied both types of PRACs in our laboratory. The ER should be larger than 20 dB in order to guarantee the QBER induced by polarization leakage is less than 1 %. For both of the polarization controllers we find that the ER can be kept above 21 dB over a period of 24 hours when maintained every 15 minutes by our automated program as shown in Fig. 6. Without periodic compensation the ER degrades gradually due to drift.

### 3.3 Time Alignment

Time alignment of the quantum streams to the classical stream is a critical issue for our QKD system. Although the quantum stream and the classical stream leave Alice’s PCB in the same 800 ps time bin (same as a bit time), their paths to the FPGA on Bob’s PCB are similar but not identical. We have designed two mechanisms for timing adjustments on Bob’s PCB by programmable delay chips, see Fig. 1, for sub-bit timing and FPGA memory for multiple bit delays. Our first step is to verify that the classical channel is active and that the copy of the classical stream routed through the XOR chips into the FPGA is also active and aligned with the classical channel. This is done in the FPGA. The next step is to have Alice continuously send quantum packets containing a known, fixed four photon pattern, all photons are encoded in the same state. Along with each quantum packet is a classical packet that heralds the arrival of the quantum packet and provides a relative time reference. Because of the high quantum losses, we build a histogram of a large number of packets to obtain the detected photon distribution within a packet. To determine the sub-bit timing alignment we step through a full bit range of delays and select the delay value of the programmable delay chip that maximizes the histogram peaks. Using that setting we then compute the difference between the time bin in which the histogram peaks appear and the bins they should be in. This yields the delay between that quantum stream and the classical stream and that value is loaded into Bob’s FPGA resulting in alignment of that quantum stream to the classical stream. We repeat this for each of the four quantum streams (encoding states). An automated program that implements this procedure is used as part of the QKD startup process.

### 3.4 Higher Order Mode Noise Reduction

**Fig. 7:**
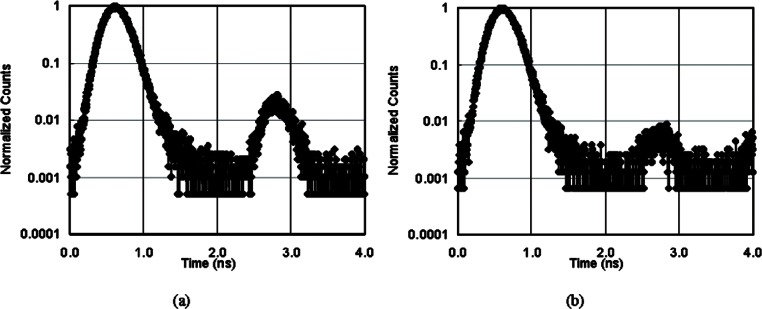
The photon detection histogram of the 850 nm quantum channel over 1 km of 1550 nm single-mode fiber (SMF28). (a) no splice; (b) ~ 40 cm of HI780 fusion-spliced at the end of the 1-km SMF28 fiber.

A practical QKD system must be able to use existing fiber infrastructure. We have devised a technique that allows 850 nm single photons to share standard telcom fiber, SMF-28, with telcom traffic. Since the cutoff wavelength of SMF-28 fiber is much longer than 850 nm, some higher order transverse modes (LP_11_ mode in our case) exist in the fiber, see Fig. 7(a). The higher order mode travels slightly slower than the fundamental mode (2.3 ns/km delay in our case). Also its polarization state is different from that of the fundamental mode. When the detection time bin is small enough (high data rates) this higher order pulse can occur in an adjacent time bin and be erroneously detected causing an increase in the QBER. By fusion splicing a short piece of HI780 fiber to the end of the telcom SMF-28 fiber it functions as a spatial filter and the higher order mode pulse is greatly suppressed [29, 30], see Fig. 7(b), allowing the 850 nm quantum channel to successfully coexist with 1550 nm traffic on standard telecom fiber.

### 3.5 System Performance and Analysis

A major limitation to the sifted-key rate is imposed by the APD. After an APD detects a photon, the avalanche process generates an electrical output signal. The device then needs a certain amount of time (dead time, Tdead) to recover to its initial operational state for detection of the next photon. During this dead time, the bias voltage across the p-n junction of the APD is below the breakdown level and no photon can be detected [35]. Moreover, in most high-speed QKD systems, the APDs operate in free-running mode and each APD works independently so when one APD is in its dead time other APDs can still detect a photon. In this case, the sifted-key rate can be calculated by [6]

$$R=2/(t_{dead}+1/R_{1})$$ (2)

where T_dead_ is 50 ns in our system and *R*_1_ is the detection count rate for each APD. *R*_1_ can be calculated by the following formula:

$$R_{1}=\mu \times v\times L_{f}\times L_{o}\times L_{c}\times L_{P}\times L_{d},$$ (3)

where μ is Alice’s mean photon number per pulse. We use μ = 0.1, but there are some discussions [31] that use μ > 0.1 resulting in a higher sifted-key rate without adverse affects on system security. The quantity *v* is the system clock rate. The photon detection efficiency, P_d_, of our APDs is 45 % at 850 nm according to the manufacturer’s specifications. We measured the optical loss (L_f_) in the transmission fiber and connectors to be −2.4 dB, the coupler loss (L_c_) to be −3 dB, the polarization beam splitter loss (L_p_) to be −3 dB (−6 dB loss for B92) and other optical losses (L_o_) to be approximately −3 dB.

**Fig. 8:**
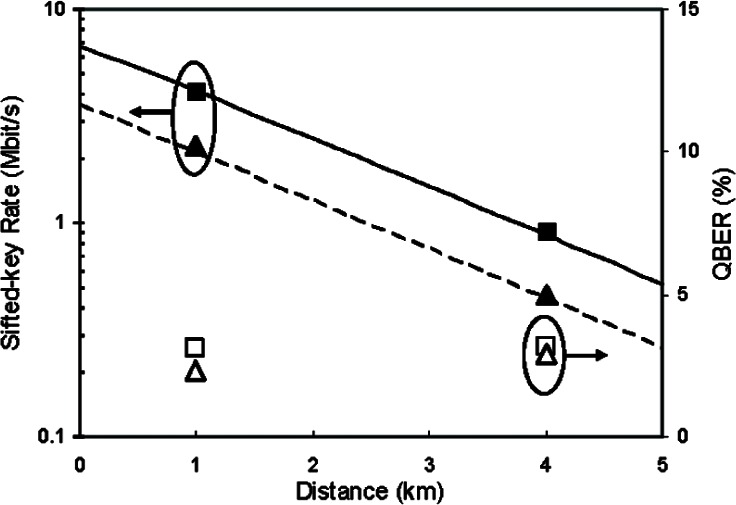
The system performance of our 850 nm QKD system using the BB84 protocol. Solid line (625 MHz) and dash line (312.5 MHz) are the calculated sifted key rate from Eq. (2). Solid squares (625 MHz) and triangles (312.5 MHz) are the measured sifted key rate. Hollow squares (625 MHz) and triangles (312.5 MHz) are the measured QBER.

The calculated and measured sifted-key rate at two transmission rates, 625 and 312.5 Mbit/s, and two fiber lengths, 1 km and 4 km, are shown in Fig. 8. The solid symbols represent the measured sifted-key rate and the lines represent the calculated values from Eq. (2). They agree well and show that this system can provide more than 4 Mbit/s of sifted key rate over a 1 km of fiber with a mean photon number of 0.1 However, due to the relative high attenuation of optical fiber at 850 nm, the sifted key rate decreases quickly as the distance increases and the sifted key rate decreases to about 1 Mbit/s at 4 km. Therefore, our 850 nm QKD system is more suitable for short distance environments.

The hollow symbols of Fig. 8 represent the measured QBER. The QBER in our system is mainly caused by the following factors: (1) APD dark count rate and light leakage; (2) Cross-talk caused by an imperfect polarization extinction ratio; (3) Timing jitter; and (4) High order mode noise. Dark counts are caused by a thermo-initiated avalanche process in the APD and unexpected photon detection. They are independent of the transmission rate and for our system are on the order of 100 per second. With proper light sealing and filtering, the counts due to light leakage are only a few tens per second. Compared to our Mbit/s detection rate, this factor is negligible. The polarization extinction ratio was measured to be between 23–28 dB during our experiments, resulting in a contribution of about 1/3 of the QBER and is independent of the transmission rate. Timing jitter is the main factor of the QBER and also limits the transmission rate. Timing jitter is mostly caused by the original optical pulse width, its jitter and the timing jitter of the APD. In our system, the optical pulse width is 800 ps (FWHM), and the jitter of the APDs is measured at about 180 ps (FWHM). We also observed APD count-dependent jitter [32] and VCSEL data-dependent jitter [33] during transmission of randomly encoded photons. Because of these jitter issues, our detection window is limited to 1.6 ns. Narrowing our detection window will result in higher QBER. High order mode noise, analyzed above, contributes about 1/3 of the QBER after our filtering technique. All of these factors together yield a QBER for our QKD system of about 2 % **~** 3 %. At a transmission rate of 625 MHz, timing jitter is the predominant QBER factor and we see little change as the distance increases to 4 km. While at 312.5 MHz, the high order mode noise and photon attenuation do slightly increase the QBER at 4 km compared to 1 km.

## 4. Cost Reduction for QKD Systems for Local Area Network

For LAN QKD systems, cost is an important concern. Our high speed 850 nm QKD system in the above section, uses 4 Si-APDs, which is the most expensive device in QKD systems. To further reduce the cost of the QKD system, we use a detection-time-bin-shift (DTBS) scheme [34]. A DTBS scheme can reduce the number of single photon detectors, by projecting the measurement bases or measured photon value into detection time-bins, rather than separate detectors. The trade-off is the system transmission clock is reduced, resulting in proportionately reduced key rates. DTBS schemes can also solve security concerns caused by detector dead-time and unbalanced detection efficiency. However, when gated mode photon detectors are used in DTBS schemes, a time-bin-shift (TBS) intercept-resend attack might undermine the security of the QKD system and counter-measures should be adopted.

In this section, we will discuss DTBS schemes, study the security issues of these schemes, especially the TBS intercept-resend attack and its countermeasures, and presents our low-cost 850 nm QKD system for LANs with only one APD based on an improved DTBS scheme.

### 4.1 DTBS Description

**Fig. 9:**
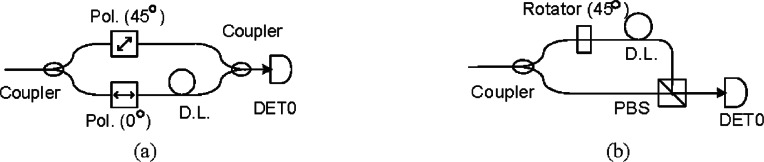
Schematic diagram of DTBS-QKD system for B92. Coupler: Passive Fiber Coupler; D.L.: Delay Line; PBS: Polarizing Beam Splitter; DET: Single Photon Detector.

The DTBS scheme was first presented by Brequet, et. al. [34], for a polarization-encoding QKD system based on the B92 protocol. We improve on this scheme by avoiding extra photon loss and extending the concept to the BB84 protocol. The DTBS scheme, which time-division multiplexes a single photon detector between two photon bases, is a trade-off between using fewer detectors and attaining higher key rates. This scheme [34], shown in Fig. 9(a), requires the single photon transmission rate to be reduced by half to allow for two DTBs. However, the second coupler in the scheme causes an additional 3dB loss and it can’t be extended to the BB84 protocol. Our improved DTBS scheme, shown in Fig. 9(b), has a simpler structure and avoids the additional coupler loss.

In the improved scheme, a passive coupler performs a random choice of measuring polarization bases and projects the results of the different bases onto a short (0° basis) or long (45° basis) delay path resulting in the photon arriving in one of two adjacent DTBs. In the short path, the polarization state of the photon is unchanged and is recorded in the first DTB. In the long path, the photon is delayed by one DTB and the polarization state of the photon is rotated by 45° and is recorded in the second DTB. The photons on these two paths are combined using a polarizing beam-splitter (PBS), thus avoiding a second coupler and its extra loss, and then fed to a single detector.

In the BB84 protocol, there are two photon values for each measurement basis, therefore, there are a number of ways to implement DTBS schemes, (I) project the measurement bases into DTBs; (II) project the photon values into DTBs; and (III) project both measurement bases and photon values into DTBs.

**Fig. 10:**
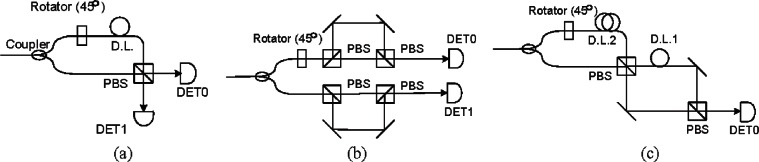
Schematic diagram of the DTBS QKD system for BB84. Coupler: Passive Fiber Coupler; D.L.1: single time-bin Delay Line; D.L. 2: double time-bin Delay Line; PBS: Polarizing Beam Splitter; DET: Single Photon Detector.

The structure of a type I BB84 DTBS scheme is shown in Fig. 10(a) and its detection photon values are shown in Table 1. After projecting the measurement bases into DTBs, the photons going through V/H basis arrive at the 1st DTB and those going through +/− 45° basis arrive at the 2nd DTB. The photon detectors are used to distinguish the photon values of “0” and “1”. We can implement this by just adding one more photon detector to the improved B92 DTBS system of Fig. 8(b). This type uses two photon detectors and each transmission clock period needs two DTBs.

The structure of a type II BB84 DTBS scheme is shown in Fig. 10(b) and detection photon values are shown in Table 2. All photons going through +/− 45° basis are detected by detector 0 and those going through V/H basis are detected by detector 1, and the different photon value of “0” and “1” arrive in different DTBs. This type uses two photon detectors and each transmission clock period needs two DTBs.

We can project both measurement bases and photon values into DTBs and use only one detector. The structure of a type III BB84 DTBS scheme is shown in Fig. 10(c) and the detection photon values are shown in Table 3. All the photons are detected by one detector. The measurement bases and photon values are distinguished only by DTBs. The “0” value photons going through V/H basis arrive in the 1st DTB and the “1” value photons arrive in the 2nd DTB. The “0” value photons going through +/−45° basis arrive in the 3rd DTB and the “1” value photons arrive in the 4th DTB. This type uses only one photon detector but each transmission clock period needs four DTBs.

### 4.2 Security Analysis of DTBS

The security of a QKD system requires that the keys must be a series of random values, which are measured randomly in two non-orthogonal bases. Even if Alice sends a series of randomly encoded photons to Bob their randomness may be compromised if the properties of the single photon detectors are not sufficiently identical. This would degrade the security of the QKD system. Three crucial security issues are: self-correlation caused by the dead-time of single photon detectors, key value imbalance and the measurement basis imbalance caused by unbalanced detector efficiencies. Since the B92 DTBS schemes and the BB84 DTBS type III scheme only use one detector they avoid these problems. QKD systems with multiple detectors do need to verify their detector properties.

#### 4.2.1 Detector Self-Correlation

Single photon detectors have a dead-time, which is the recovery time following each detection event. The dead time for a Si-APD in our 850 nm QKD system is 50 ns. During the dead time, the bias voltage of the detector is below the breakdown level and no photon can be detected [35]. The detector dead-time makes detectors temporarily unavailable, which can result in repeating detector firing order. For example, in a conventional B92 system, two detectors are used to detect “0” and “1”, respectively. Once one detector has been fired by a photon, it becomes unavailable for the duration of its dead time. In a high photon transmission rate system there is a high probability that the other detector will fire before the first detector recovers. If this sequence of one detector being dead while the other detector fires continues, it results in strings of 1010…. Runs of such strings reduce the randomness of the keys and degrade the security of the QKD system. Conventional BB84 systems suffer from the same problem. This dead-time induced self-correlation problem has been discussed previously [36, 37].

In the BB84 DTBS type II scheme each photon detector measures both the “0” and “1” values, so detector firing order does not effect the randomness of measured values. A type I BB84 DTBS scheme is still susceptible to the self-correlation problem.

#### 4.2.2 Key Value Imbalance

Since the keys are random, this means the values “0” and “1” occur with equal probability, 50 %. However, in conventional systems, separate photon detectors are used for different photon values (0 or 1) and its difficult to build all photon detectors with identical detection efficiency. A detector with higher efficiency would fire more frequently than one with lower efficiency. This unbalanced characteristic would cause key values to skew more towards one of the two values and undermine the randomness of keys. In the BB84 DTBS type II scheme each photon detector measures both the “0” and “1” values, so detector firing order or firing frequency does not affect the randomness of measured values and avoids this security concern. A type I BB84 DTBS schemes is still susceptible to a key imbalance problem.

#### 4.2.3 Measurement Basis Imbalance

Random selection of the non-orthogonal measurement bases is crucial for the security of distributed keys. Unbalance detection efficiency might skew the measurement basis used on arriving photons and undermine the randomness of the measurement bases.

In the BB84 DTBS type I scheme each detector measures both non-orthogonal bases, so detector firing order or firing frequency does not affect the randomness of the basis measurement and avoids this security concern. The type II BB84 DTBS scheme is still susceptible to a basis imbalance problem.

Table 4 summarizes these security concerns in these schemes caused by the detector dead-time and unbalanced detector efficiency. The conventional schemes suffer from all these security concerns. It shows the B92 DTBS scheme and type III BB84 DTBS scheme avoid all these security problems. Type I and type II of BB84 DTBS schemes can suppress only some of these security problems.

#### 4.2.4 TBS Intercept-Resend Attack and its Countermeasures

Some DTBS schemes are also vulnerable to the TBS intercept-resend attack when single photon detectors operate in a gated mode. Some single photon detectors, such as InGaAs APDs, can only work in a gated mode, in which the detectors only can detect photons in a specified time window. DTBS systems with single photon detectors operating in free-running mode, such as Si-APD or SSPD, are not susceptible to this attack.

**Fig. 11:**
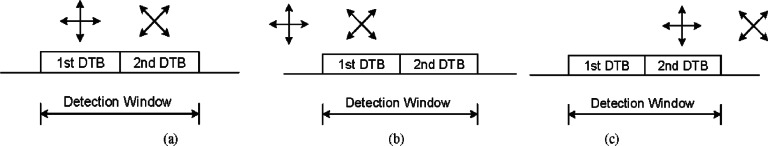
A TBS intercept-resend attack on DTBS system with gated photon detectors. (a) without a time-shift (b) one time-bin advanced shift (b) one time-bin delayed shift.

A TBS intercept-resend attack occurs when Eve intercepts a photon, measures the photon, and then sends a new photon in its place, encoded with the measured value but the opposite basis, in either an earlier or a later time bin. Because gating the detector forces an inactive detection period, Eve can time photon arrivals so that she can force Bob to use a known measurement basis or take no measurement at all. We use a type I BB84 DTBS scheme here as an example. If the gated detection window is set to two DTBs, as shown in Fig. 11(a), Eve can intercept a photon, measure it with H/V basis, and then send another photon using that measured value in the +/− 45° basis one time bin earlier. The DTBS optics in front of the detectors determine the measurement basis for arriving photons and will process the photons whenever they arrive, independent of the detector gating. If Bob measures the photon in the H/V basis, it will arrive a time bin too early and not be detected. If Bob measures the photon in the +/− 45° basis, as Eve has sent it, it would arrive in the 1st time bin and be detected as being measured with H/V basis, the same as Eve actually measured it, as shown in Fig. 11(b). During sifting, Bob tells Alice the basis it used to measure that photon and Alice replies to Bob letting him know if it was correct. Eve can obtain this information from the classical channel and know which photons were measured correctly and which ones to discard. Since Eve forced Bob to measure the photon in the same basis she measured it, Eve will not induce any errors in Bob’s sifted bits and Eve will know the sifted key without being detected. By the same method, Eve can measure intercepted photons in the +/− 45° basis and re-send another photon in the H/V basis one time bin later forcing Bob to tally the measurement as a +/− 45° basis or not at all, as shown Fig. 11(c). In this way, Eve can randomize the measurement basis she uses, making communication between Alice and Bob seem normal with no unbalances detected in the measurement of values as well as the QBER.

**Fig. 12:**
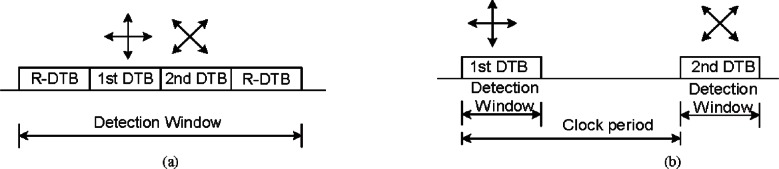
Countermeasures for TBS intercept-resend attack. (a) Guard DTB; (b) one DTB per detection window. DTB: Detection time bin. R-DTB: Guard detection time bin.

There are two countermeasures to avoid the TBS intercept-resend attack: one is using guard time bins and the other is to use only one time bin per detection window. We use a type I BB84 DTBS scheme as an example. Guard time bins, as shown in Fig. 12(a), extend a detection window to contain two additional DTBs. The Guard DTBs work as a monitor to check for any abnormal counts that a TBS intercept-resent attack may cause, but its main drawback is that dark counts would increase as the detection window broadens. Another countermeasure is using only one DTB per detection window, as shown in Fig. 12(b), where the delay between the two DTB is the same as the gating clock period. In this approach, no increase in dark counts occurs and there is no change in photon arrival times that Eve can use that will force Bob to make a known basis measurement. A drawback of this approach is that we must reduce the transmission clock rate by half.

### 4.3 System Configuration

**Fig. 13:**
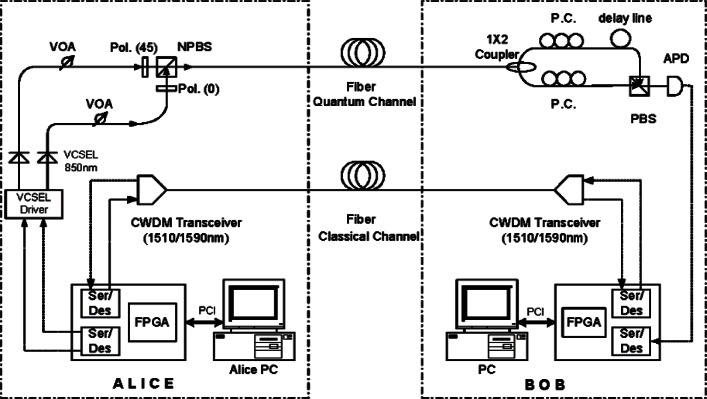
Schematic diagram of our B92 DTBS-QKD system; VCSEL: Vertical-Cavity Surface-Emitting Lasers; Pol.: Polarizer; VOA: Variable Optical Attenuator; NPBS, Non-polarizing Beam Splitter; P.C.: Polarization Controller; FPGA; Custom printed circuit board controlled by a field-programmable gate array; PCI: PCI bus; PBS: Polarizing Beam Splitter; Solid line: Optical fiber; Dotted line: Electric cable.

We modified our 850 nm QKD system (Fig. 3) to incorporate DTBS in Bob for the B92 protocol, as shown in Fig. 13. We have decreased Alice’s quantum transmission rate from 625 MHz to 312.5 MHz to allow for two DTBs.

The arriving photons at Bob are randomly selected by a 50/50 passive fiber coupler and fed into a long or short path. Polarization controllers are used to recover the polarization state and add another 45° polarization rotation in the long path. The optical delay between the long and the short paths is 1.6 ns, or one DTB. All photons are then passed or reflected by a PBS and then detected by a Si-APD. For each transmission clock period (3.2 ns), the “0” photons arrive at the detector in the first DTB and the “1” photons arrive in the second DTB.

For our DTBS implementation, only one quantum stream needs to be aligned, rather than two for normal B92, since BTBS combines the two channels into one. During sifting, Bob and Alice use the transmission clock time bin of detected events, not the DTB, otherwise the QKD protocol remains unchanged.

### 4.4 System Performance and Analysis

Two configurations are used in our measurements of our DTBS QKD system: a back-to-back configuration (two 2 meter patch-cords of SMF-28 and HI-780 are used for the classical and quantum channels, respectively) and a 1.1 km configuration (two 1.1 km SMF-28 fibers are used, one for the classical and one for the quantum channel, and Bob’s end of the quantum channel is fusion-spliced to a 40 cm HI-780 fiber).

**Fig. 14:**
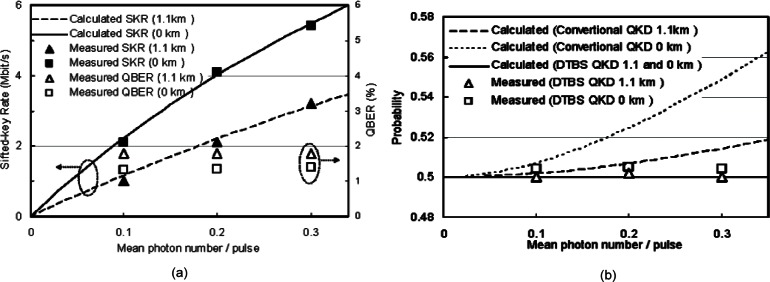
The system performance of our B92 DTBS QKD system in a back-to-back and a 1.1 km configurations. (a) Sifted Key Rate (SKR) and QBER (b) Probability that neighboring two bits are different.

For each configuration, we measured the sifted-key rate and the QBER, two important performance metrics for QKD systems. In Fig. 14(a) we plotted the measured data as a function of the mean photon number, μ, along with the theoretically calculated sifted-key rates from Eq. (2), and we see that the measured data agrees well with the theoretically calculated values. Our conventional BB84 QKD system achieves more than 4 Mbit/s sifted key rate, while our B92 DTBS QKD system achieves more than 1 Mbit/s sifted key rate at μ = 0.1 over 1.1 km of fiber. Because we must decrease our transmission clock rate by half for DTBS and we’re using B92 instead of BB84, we expect our DTBS sifted key rate to be about 25 % of the BB84 results. However, the cost of the system is reduced significantly since it needs only one APD instead four for BB84. QBER is mainly due to dark counts, polarization leakage and timing jitter, as discussed in (polarization compensation) Sect. 3 above. Using DTBS does not cause any increase in the QBER compared to a conventional QKD system.

We use the next bit probability as a metric for the randomness of keys, which is the probability that two neighboring bit values are different. For random keys, the next bit probability is 0.5. Fig. 14(b) shows the calculated next bit probability for traditional QKD and DTBS QKD systems [36, 37] and the measured results from our DTBS QKD system. In conventional QKD systems, the next bit probability may not be 0.5 at high data rates due to the dead-time introduced self correlation, while in DTBS QKD systems the next bit probability remains at 0.5 as required by the protocol. Because only one APD is used in the system, unbalanced detection efficiency problems are avoided. Furthermore, the APD used in our system operates in the free-running mode, avoiding any threat from the TBS intercept-resend attack.

## 5. High-Speed QKD Systems for Metropolitan Area Network

For longer distance QKD systems, the wavelength of the quantum signal needs be in the 1310 nm or 1550 nm bands, where the telecom fiber loss is lowest. A QKD system that can operate at distances of up to 50 km or 100 km is applicable for use in a Metropolitan Area Network (MAN). WDM and erbium-doped fiber amplifier (EDFA) technology are widely used in current optical fiber network and the noise they induce in the 1550 nm band is too high to allow single photon transmission in that band of the same fiber. This leaves the 1310 nm band as a good compromise for single photon transmission that can share a fiber with existing 1550 telcom traffic via WDM.

Among the single photon detectors available for the 1310 nm band, InGaAs avalanche photodiode (APD) [38], superconducting single-photon detector (SSPD) [39] and up-conversion detector using Si-APDs [40] are used to implement high-speed QKD systems. Recently, a self-difference technique was developed for InGaAs APDs that suppresses the afterpulse noise, and it has been successfully applied to a GHz QKD system [41]. The InGaAs APD has about 10 % detection efficiency, but it still has about 6 % afterpulse probability which would contribute an extra 3 % to the QBER of a QKD system. SSPDs can operate in the free-running mode and their response time can be less than 100 ps. However, SSPDs are expensive and need to be operated at 4 °K. Si-APDs are low cost, operate at room temperature and have the highest peak detection efficiency among these detectors—70 % around 650 nm, but they don’t operate at wavelengths longer than 1000 nm. To alleviate this limitation we implemented an up-conversion detector that transforms 1310 nm single photons into 710 nm photons that will work well with Si-APDs.

### 5.1 Low Noise Up-Conversion Technology

**Fig. 15:**
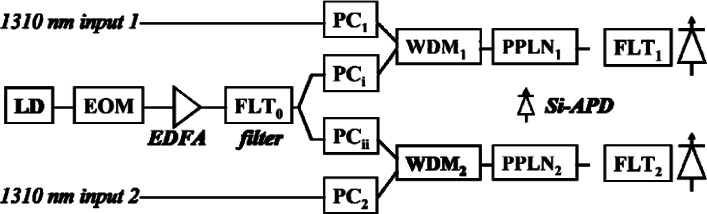
The configuration of our 1550 nm pump up-conversion detectors. LD: Laser diode; EOM: Electric-optic modulator (LiNbO_3_); EDFA: Erbium-doped fiber amplifier; FLT: Optical filter; PC: Polarization controller; WDM: Wavelength-division multiplexer for 1310 nm and 1550 nm; PPLN: Periodically-poled LiNbO_3_ module.

Our up-conversion detector structure is shown in Fig. 15. The 1557 nm CW laser diode output is modulated to a pulse stream and then amplified using an EDFA. An optical filter, FLT_0_, with the FWHM) of 7 nm is used to suppress the noise of the EDFA. This filter is important because the optical noise between 1000 nm and 1300 nm can induce a large amount of dark counts and the WDM before the Periodically-poled LiNbO_3_ (PPLN) may not be sufficient to suppress this noise. After the FLT_0_, the 1557 nm pulse is divided into two streams by a 50:50 coupler to function as a pump for our two QKD quantum streams at 1306 nm. After polarization control is applied, the 1306 nm QKD signals and the 1557 nm pump are combined by the WDMs, sent to the PPLNs (HCP Photonics) where they are up-converted to 710 nm outputs and then further filtered and finally detected by the Si-APD [24]. The filtering of the signal after the PPLNs differs due to a manufacturing error. The output of PPLN_2_ is coupled to a 700 nm single mode fiber, which cuts off the strong 1550 nm pump light and is passed to FLT_2_. FLT_2_ contains two filters: a 20 nm band-pass filter (Omega Optical, Inc: 3RD700 – 720) and a short-pass filter (Omega Optical, Inc: 3RD730SP). This combination of filters helps to attenuate the light between 730 nm to 1000 nm by more than 80 dB. The output of PPLN_1_ is coupled to a standard single-mode fiber and the strong 1557 nm pump is not attenuated sufficiently FLT_1_ uses a different short-pass filter (Omega Optical, Inc: 725ASP) that can sufficiently attenuate light between 730 nm and 1600 nm but has a larger loss around 710 nm than the 3RD730SP in FLT_2_. Furthermore, the optical pulse is broadened by nearly 100 ps because the wrong fiber pigtail has a multi-mode propagation of the 710 nm light. In Table 5, we list the transmittance parameters of the two up-conversion detectors. As shown in the table, the internal quantum conversion efficiency of the two PPLNs is almost 100 %. However, the coupling loss is significantly larger than those in [40] and therefore degrades the overall detection efficiency.

**Fig. 16:**
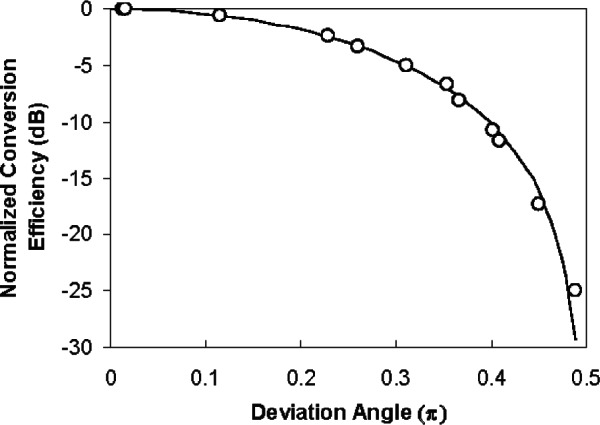
The PPLN_1_ normalized conversion efficiency as a function of deviation angle of the input 1306 nm signal. The deviation angle is the angle between the given polarization state and the one by which the conversion efficiency is maximal. The polarization state and the deviation angle are described in Jones space. The polarization sensitivity of PPLN_2_ is similar.

PPLN up-conversion is known to be polarization sensitive. If the polarization extinction ratio of the PPLN is sufficiently high, it can be used as a polarizer. In Fig. 15 we show the dependence of the PPLN_1_ conversion efficiency on the deviation angle of the 1306 nm input polarization state. Similar results were obtained with PPLN_2_. The deviation angle is the angle (in Jones space) between the given input polarization state and the one by which the conversion efficiency is maximal. We also compared the measurement results with a cos^2^(x) curve, which is an ideal polarizer. The curve agrees well with the measured data and we believe that the slight difference is caused by the measurement uncertainty of the polarimeter. As shown in Fig. 16, the polarization extinction ratio of the PPLN is not less than 25 dB. Therefore, we used the PPLN as a polarizer in our B92 QKD system, saving a 1 dB loss that a separate polarizer would add.

In previous work, the PPLN are pumped by continuous-wave (CW) light. As shown in Fig. 14, our pump is formatted to a pulse train that is synchronous to the signal clock. The dark count rate and the conversion efficiency of two up-conversion detectors are shown in Fig. 16. The 1306 nm quantum signal is a 625 Mbit/s random encoded pulse train and the 1557 nm pump is a 625 MHz pulse train and the two are synchronized. The FWHM of the signal and the pump are 220 ps and 620 ps respectively.

**Fig. 17:**
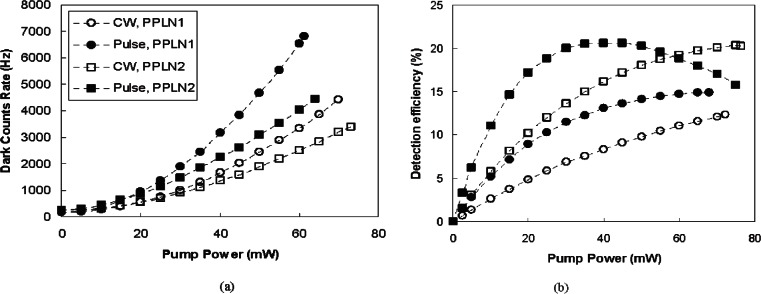
The dark count rate (a) and detection efficiency (b) as a function of pump power at the PPLN input. Four cases are studied: two up-conversion detectors, each pumped by CW and pulse.

As shown in Fig. 17, the pulse pump generates more dark counts than the CW pump at a given average pump power because the peak pump power of the pulse is higher than that of the CW and pump power has a large amount of high order components. We refer to pump power as the average power of the pump. The pulse pump needs less power than the CW pump to achieve a given detection efficiency. The overall result is that the pulse pump can achieve a given detection efficiency with less dark counts compared to the CW pump. For example, the PPLN_2_ detection efficiency using the pulse pump at 16 mW is 15 % and the dark count rate is 660 counts/s. The CW pump needs 35 mW of power to achieve a 15 % detection efficiency and incurs a dark count rate of 1100 counts/s. Consequently, using a pulse pump effectively reduces the dark count and power compared to a CW pump. Fig. 16 and Table 5 also suggest that we achieved almost 100 % internal up-conversion efficiency with a pulse pump because the pump pulse is significantly wider than the signal pulse.

By using strong pulse light at 1550 nm to pump signals at 1310 nm and applying proper filtering, we achieved substantially lower dark count rates in our up-conversion detector. When the pump power is set at 40 mW, the dark count rate is 3200 counts/s with the PPLN_1_ detector and 2200 counts/s with the PPLN_2_. Such dark count rates are nearly two orders of magnitude lower than that given by the 1310 nm pump scheme [40].

### 5.2 System Configuration

**Fig. 18:**
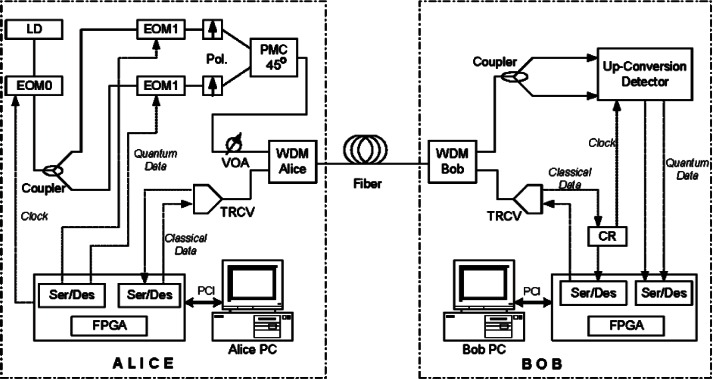
The B92 polarization coding QKD system. LD: Laser diode; EOM: Electric-optic modulator (LiNbO_3_); PC: Polarization controller; PMC–45°: Polarization maintaining combiner that combines two light signals that are separated by 45 degrees; VOA: Variable optical attenuator; WDM: Wavelength-division multiplexer; SMF: Standard single-mode fiber; TRCV: Optical transceiver; CR: Clock recovery module; FPGA: Custom printed circuit board controlled by a field-programmable gate array; PCI: PCI connection; Up-conversion detector: See Fig. 2; Dotted line: Electric cable; Solid line: Optical fiber.

We applied our 1550 nm pump up-conversion detector to our B92 polarization based QKD system shown in Fig. 18, with a quantum channel transmission rate of 625 MHz at 1306 nm. At Alice, 1306 nm CW light is first modulated to a 625 MHz pulse train and then evenly split into two polarization channels and further modulated by a quantum data sequence (625 Mbit/s random bit sequence) generated by Alice’s PCB. The FWHM of the quantum data pulse is 220 ps. At most, only one quantum stream is active in any given time bin. With a polarizer followed by a 45-degree polarization maintaining combiner, the two streams are combined for transmission through the quantum channel with their polarization states being 45 degree away from each other. After being attenuated to the mean photon number of 0.1 per bit, the 1306 nm quantum signal is combined with the classical channel via a WDM. Both the quantum and the classical channels are transmitted to Bob through a standard single-mode telcom fiber. At Bob, another WDM is used to separate the quantum and the classical channel. The quantum channel is processed by a 1550 nm pulse pump up-conversion detector shown in Fig. 15 and the detection results are sent to Bob’s PCB. Bob’s PCB extracts Alice’s clock from the classical channel and uses that clock to generate the pump pulse train. The remaining stages of the QKD protocol are the same as for our 850 nm system and are carried out on our PCB and the associated computers.

### 5.3 System Performance and Analysis

Our PCB currently has a QKD distance limitation of about 15 km, because of the limited amount of memory allocated to store the quantum stream during the round trip time to Bob and back for sifting. When this memory becomes full, Alice stalls the quantum stream until the sifting messages return from Bob and allows Alice to free that memory. This results in a lower sifted-key rate since there is no quantum stream during this stall period. For example, if the distance between Alice and Bob is 50 km, or 250 μs through fiber, the quantum stream is only active for 75 μs during that 250 μs period. Since in this case, Alice is only sending quantum data about 30 % of the time, the resulting key rate would be only 30 % of it potential if the memory were large enough so that Alice wouldn’t stall. So instead of using the measured sifted-key rate from our system, we calculate and effective key rate that represents our QKD system’s potential capacity. Our effective sifted-key rate is calculated by the number of keys reported by Bob divided by Alice’s active time, which is the product of the bit period (1.6 ns) and the total number of bits sent by Alice during that active time.

The quantum channel (1310 nm) and the bi-directional classical channel (1510 & 1590 nm) share (WDM) a single standard telecom fiber in our 1310 nm system, so there is concern that the quantum channel may suffer noise from the classical channel. The classical channel could induce dark counts in two ways: transceiver noise and nonlinear effects. First, the transceiver emits a certain amount of optical noise around 1310 nm. Some of this noise will leak into the PPLNs and then be up-converted to 710 nm. Second, the 1510 nm and 1590 nm light generate photons around 1310 nm via the anti-Stokes process and these non-linearly induced 1310 nm photons will be up-converted to 710 nm in PPLN. In Fig. 18(a), we show the extra dark count rate induced by the classical channel at various distances. We first measure the dark count rate when one or both of the classical transceivers are on, and then subtract the dark count rate measured when both transceivers are off. The photon leakage can be evaluated by the extra dark counts in the back-to-back (0 km) connection while the nonlinearly induced dark photon effect will vary over the transmission distance.

**Fig. 19:**
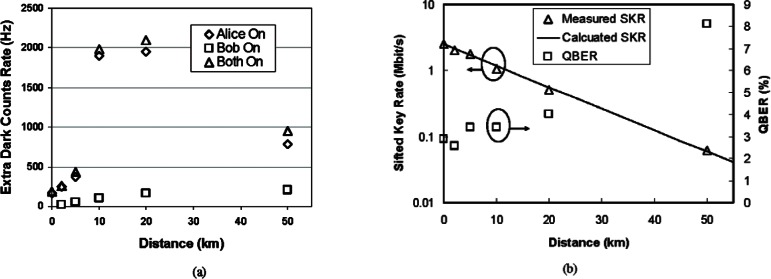
The extra dark count rate induced by the classical channel in PPLN_1_ detector in three cases: Square, only transceiver at Alice is on; Diamond, only transceiver at Bob is on; Circle, both transceivers are on. The PPLN_2_ detector exhibits similar behaviors. (b) The system performance of the B92 polarization-based QKD system with the 1557 nm up-conversion detector.

As shown in the Fig. 19(a), the photon leakage noise is small and the dark count is mainly induced by the nonlinear anti-Stokes process, particularly from the 1510 nm light propagating from Alice to Bob (forwards anti-Stokes). The forwards anti-Stokes is stronger than the backwards one (the backwards anti-Stokes noise generated by the 1590 nm light propagating from Bob to Alice) because the 1510 nm light is 80 nm closer to the 1310 nm than the 1590 nm light. The dark counts induced by the forwards anti-Stokes increases over distance up to 20 km, because in this region the accumulated anti-Stokes process wins over the accumulated fiber loss. After 20 km, the dark count rate reduces as the accumulated fiber loss overtakes it. The 1510 nm light is attenuated so that less anti-Stokes noise is generated after 20 km. The anti-Stokes noise generated before 20 km is also attenuated by the fiber loss. By comparison, the dark counts induced by the backwards anti-Stokes noise also increases over distance but in this case it saturates after 20 km: both the 1590 nm classical light and backwards anti-Stokes noise are sufficiently attenuated by the fiber loss and therefore almost no additional anti-Stokes is reflected back as the fiber length increases beyond 20 km. In general, the classical channel induces negligible dark counts into the QKD system, particularly from 1510 nm. A longer wavelength transceiver would greatly help to reduce the dark count, but care must be taken to keep it within the standard telecom band.

The system performance is shown in Fig. 19(b). During our measurements, the pump power was fixed at 40 mW. The sifted-key rate is 2.5 Mbit/s for a back-to-back connection, 1 Mbit/s at 10 km, and 60 kbit/s at 50 km. The QBER is approximately 3 % back-to-back, remains below 4 % up to 20 km, and reaches 8 % at 50 km. The finite extinction ratio of modulator and timing jitter of the system induces a background QBER of approximately 2.5 % and the rest is from dark counts generated by both the pump light and the classical channel, as we described earlier. We also calculated the theoretical sifted-key rate and QBER and they agree well with the measured results. Although we fixed the pump power close to the maximum up-conversion efficiency, the QBER remains small until 20 km due to the low dark count rate of the 1550 nm up-conversion detector.

## 6. QKD Networks

A quantum network links QKD systems together to produce shared secrets, and it is attached, or embedded, into conventional communication networks that send secure messages. We have developed several point-to-point QKD systems applicable for LANs (< 5 km) and MANs (< 50 km). Integrating these QKD systems into a quantum network that supports traditional security protocols and uses the existing network infrastructure is an important step towards the practical deployment of these systems.

There are two types of QKD networks, passive and active. Passive networks use passive optical components (e.g., the optical coupler) to implement multi-user connectivity. Passive networks can realize multi-terminal communications simultaneously, or “broadcast” from one node to multiple nodes. Several groups have successfully demonstrated a passive QKD network [42, 44]. However, in a passive network, the photons (and hence the bits that they represent) are split by couplers according to their coupling ratio and distributed proportionally to each node, resulting in a greatly reduced key rate between each node. The second type adopts active optical components, such as optical switches, to dynamically control the communication path. This type is similar to current switched optical communication networks, and establishes a reconfigurable QKD link. The system switching time and the influence of the active optical devices on the QKD system are the main factors used to evaluate this type of network. Optical switches have been investigated in QKD systems [45], and demonstrated in one [46] but their security protocol implementation didn’t support a one-time pads cipher.

We have demonstrated a complete 3-node, active QKD network controlled by commercial MEMS optical switches. The system operates at a 1.25 Gbps clock rate and can provide more than one Mbps sifted-key rate over 1 km of optical fiber. As part of this QKD network, we have developed a high-level QKD network manager that provides QKD services to security applications. These services include managing the QKD network, and demultiplexing and synchronizing the secure key stream. To demonstrate the speed of our QKD system, we have developed a video surveillance application that is secured by a onetime pad cipher using keys generated by our QKD network and transmitted over standard internet IP channels.

### 6.1 System Configuration

**Fig. 20:**
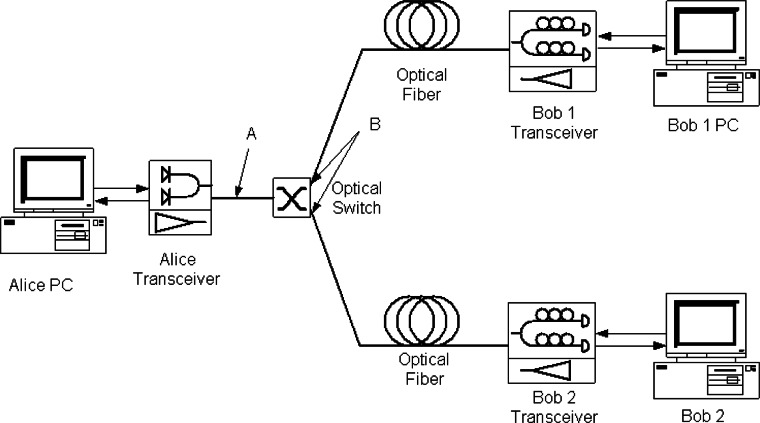
Configuration of active 3-node network. Two separate optical fibers are used for the quantum and classical channels in each link.

Our 3-node QKD network uses our 850 nm QKD system configured for B92 (and capable of supporting BB84 with the addition of 2 APDs & 2 VCSELs), and is shown schematically in Fig. 20. To connect our QKD nodes we’ve added a pair of MEMS optical switches, one for the bi-directional classical channel (1510 & 1590 nm) and the other for the quantum channel. Both channels use standard telecom fiber (SMF-28), but the quantum channel has a short length (**~** 20 cm) of HI-780 fiber fusion spliced to the end to remove the higher order mode components generated in the SMF-28.

### 6.2 Results and Analysis

The sifted-key rate and QBER are the criteria used to evaluate QKD system performance. Switching time, the time used to establish the QKD connection between Alice and Bob, is an additional criterion to evaluate a QKD network.

The sifted-key rate is determined by the factors listed in Eq. (3) of Sec. 3.5, with an additional insertion loss factor for the switch that we measured to be about 1 dB. The other factor values are the same as listed in section 3.5, except the fiber loss here is 2.3 dB instead of 2.4 dB. We measured the sifted key rate based on three different settings. In setting 1, the mean photon number, μ is set to 0.1 before the optical switches, point A in Fig. 20. In setting 2, μ is set to 0.1 after the optical switches, at point B in Fig. 20. In setting 3, μ is set to 0.1 at Alice’s output without the switch, just a direct point-to-point link. The results are shown in Table 6. We obtain the same key rate with a switch, when μ = 0.1 at point B, as without a switch. Also we see that with μ = 0.1 at point A, we do indeed suffer the expected 1 dB loss from the switch. Thus some performance advantage can be gained from using a LAN star configuration where the switch can be contained solely in Alice so that μ = 0.1 can be set after the switch. In a more general NxM LAN configuration the switches could not reside at a single central node and thus μ = 0.1 would need to be established at the output of each Alice, before the switches.

Our results also show that the links to Bob 1, in which the standard 1550 nm single-mode fiber SMF-28 is used, induces more photon loss. This is due to the fact that SMF-28 cannot provide single mode transmission at 850 nm and those photons in the higher mode are filtered by the short length of HI-780. By comparison, the link to Bob 2 uses HI-780 throughout and there is no photon loss due to the filtering of higher-mode photons. For a 1 km of transmission length, the photon loss due to the higher mode transmission and subsequent filtering is less than 1 dB, as shown in Table 6.

We measured the polarization extinction ratio, timing jitter and QBER in the two links, both with and without the optical switch as shown in Table 7. The results indicate that the QBER does not change significantly when the switch is added. Therefore, the switch can be regarded as transparent for polarization-encoding QKD without significantly adding to the QBER. The dark count rate of Si-APD is less than 100 counts/s, which is significantly less than the sifted key rate of over one Mbps. It can be concluded that polarization leakage and timing jitter are the main error factors.

**Fig. 21:**
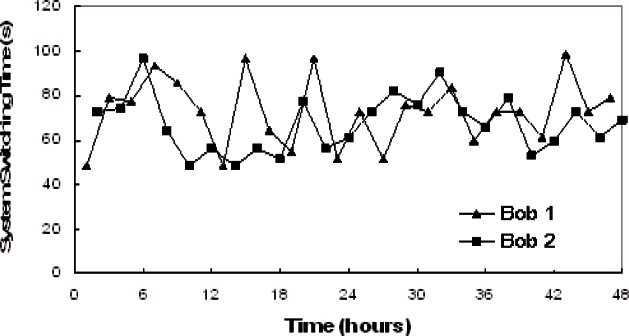
Measured switching time. The switching time is measured at each switch operation, which is performed every hour over a 48-hour period.

In an active QKD network, we define switching time as the time taken to establish secure key transmission after the switching signal is received. The switching time includes the times of four subsequent operations: optical switching, polarization recovery, time alignment and protocol initialization. The switching time of the optical switches is less than 1 ms. Polarization recovery, on the other hand, is relatively long. During polarization recovery, multiple photon samples are collected by each detector and are then used as feedback to adjust the piezo-driving polarization controller (see Sec. 3.2). Although the response time of the polarization controllers is as small as 100 μs, the time required to collect enough photons for each feedback sample is about 50 ms. Moreover, the number of samples vary before the optimum points are found, details have been described elsewhere [47]. Polarization recovery times are variable and range from several seconds up to 50 seconds. Timing alignment is needed to compensate for delay differences between the classical and the quantum channels. Our automatic timing alignment takes approximately 5 seconds per quantum channel. The protocol initialization includes starting the quantum transmission, accumulating sufficient sifted key to invoke error reconciliation and privacy amplification, and then waiting for that to produce the first few Mbits of secure key. This depends on the speed of both Alice’s and Bob’s computers as well as the quantum transmission rate. Protocol initialization time was measured at approximately 40 seconds. Our switching time is therefore approximately 1 **~** 2 min with an average of 69 s, as shown in Fig. 21 which plots hourly measurements over a 48 h period.

### 6.3 Quantum Network Management

**Fig. 22:**
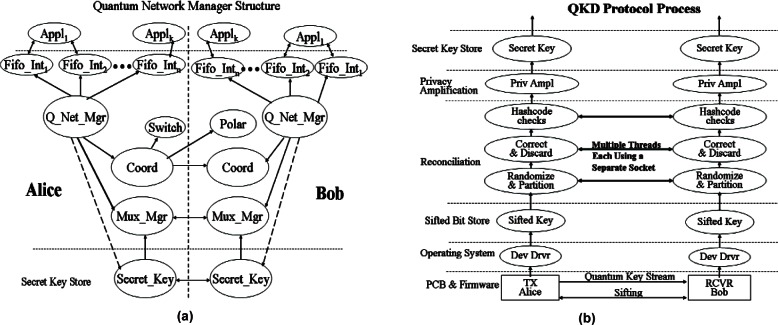
(a) Quantum Network Manager Structure (b) NIST QKD protocol flow.

A practical QKD network is a scarce resource and requires a utility program that coordinates the operations of all QKD nodes, such as switching, polarization recovery, timing alignment and protocol initialization, as well as provides services to upper layer security applications such as routing availability and secure key demultiplexing and synchronization. We developed a quantum network manager that performs these functions through various sub-managers, see Fig. 22(a). The quantum network coordination operations have been discussed above and are carried out by the coordinator sub-manager; here we discuss the necessary quantum services. Since security applications aren’t aware of the quantum network topology, they need the ability to determine if two nodes are connected via the quantum network and if that connection is possible. This could be a routing availability query to the quantum network manager using IP addresses (or DNS names) of the communicating nodes. Another necessary service is supplying a demultiplexed and synchronized secure key to a number of independent security applications. QKD produces a pool of identical secure keys at the two ends of a QKD link that are an ordered set of bits. Demultiplexing and synchronization splits this pool into independent streams such that the same amount and the same sequence of secure keys are allocated to each stream at the two ends of a QKD link. Not only should there be independent key streams for each application, but there should be multiple streams for each application. For example, a security application, such as IPsec, could have two streams for each security association. One stream for the initiator and the other for the responder (i.e., send & receive). Thus, messages going in opposite directions would not have to deal with reserving and synchronizing encryption keys because the send messages would have their own key stream as would the receive messages. Our quantum network manager provides this service, via the Mux sub-manager, as independent FIFO interfaces, where each stream is a separate FIFO and all FIFO reads are destructive. Parameters passed to the manager when opening a new stream contain an ID tag and indicates the size of the key and the frequency of re-keying. The ID tag is necessary to identify the same stream at both ends of the link. The other two parameters provide information for buffer allocation and scheduling.

**Fig. 23:**
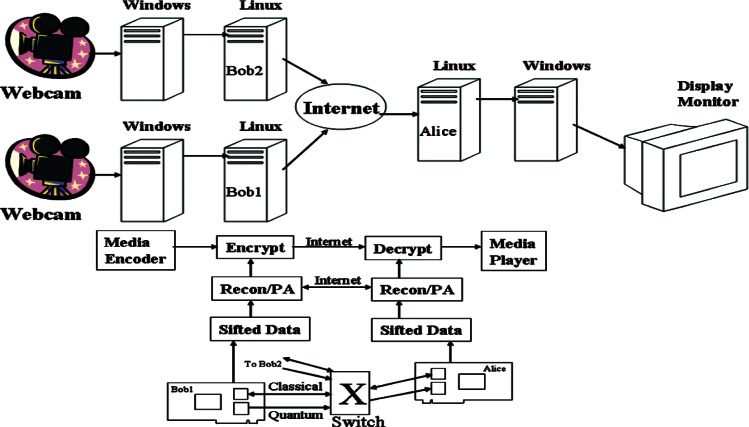
Surveillance application secured by a QKD network and a one-time pad cipher.

A one-time pad cipher is a proven secure encryption method. It requires one bit of key for each bit of data to be encrypted. Thus for high message traffic or streaming data, a high-speed stream of encryption keys is required. A benefit of a one-time pad cipher and the machinery necessary to produce the associated high key rate is the simple encryption/decryption algorithm, a bit-by-bit XOR operation of the data stream with the key stream, adding little overhead to an application. To demonstrate the performance of our QKD network we have developed a secure video surveillance application that uses a one-time pad cipher to encrypt streaming video in real-time, as shown in Fig. 23. Two Bobs, at two different locations, are each equipped with a monitoring video camera, and are linked to Alice, who resides at the surveillance station, through a switched QKD connection and the internet.

Our surveillance application uses commercial web-cams and an open source media encoder and player, all of which run on standard Windows based PCs. Each webcam output is processed by the media encoder and sends a UDP video data stream to its attached Bob (Linux) machine. Only one Bob (i.e., Bob 1 or Bob 2) at a time is active and connected to Alice through the switch. Our encryption application, running on the active Bob, receives the video stream as well as a stream of privacy amplified keys from its local QKD protocol flow, see Fig. 22(b), and performs a one-time pad encryption on the video stream. The now encrypted video stream is sent over the internet to Alice, also a Linux machine. Our decryption application, running on Alice, receives the encrypted TCP video stream as well as the matching synchronized stream of privacy amplified keys from its local QKD protocol flow. It then uses the key stream to decrypt the video stream and sends the clear text video as a UDP stream to its attached Windows based PC, which is running the media player that displays the video on the PC monitor. The result is continuous video, although delayed by a few seconds, being display from the webcam. When a user at Alice’s monitor chooses to switch the video between Bob 1 and Bob 2, the currently active Bob continues to send encrypted video to Alice while its QKD protocol flow is terminated. The inactive Bob’s QKD protocol flow is started up. The active Bob will continue to send encrypted video until his key store is depleted or the inactive Bob’s starts to generate key. Then the active designation is switched and Alice waits for its encrypted video stream to begin from the newly active Bob. Alternatively we could terminate the active Bob’s encrypted video immediately and conserve his secure key pool for when we switch back to him. We could then use whatever key pool exits at the inactive Bob to start sending secure video immediately while he begins to generate new key material.

## 7. Conclusion

We have presented two complete high-speed fiber-based QKD systems that were developed at NIST. Our 850 nm QKD system is a short distance system and was designed using a number of low cost features that make it suitable for use in LANs. It can achieve a sifted key rate of more than 4 Mbit/s over 1 km of fiber. Our 1310 nm QKD system is a longer distance system and more suitable for use in MANs. It can achieve a sifted key rate of more than 60 Kbits/s over 50 km of fiber. We have also presented a switched quantum network, along with the software utilities needed to control the quantum network and provide services to upper layer security applications. A secure video surveillance application was developed that demonstrates the performance of our QKD network. It generates a one-time pad cipher in real-time to encrypt live streaming video and the choice of which stream to view can be switched on demand. Our intention is to show the current sophistication of QKD systems and their feasible use in quantum networks that are capable of being integrated into today’s networking infrastructure to provide cryptographic support.

## Figures and Tables

**Table 1:** Detection values of type I DTBS BB84 scheme

	1^st^ DTB	2^nd^ DTB
DET 0	‘0’ (V/H basis)	‘0’ (+ / − 45° basis)
DET 1	‘1’ (V/H basis)	‘1’ (+ / − 45° basis)

**Table 2:** Detection values of type II DTBS BB84 scheme

	1^st^ DTB	2^nd^ DTB
DET 0	‘0’ (+ / − 45° basis)	‘1’ (+ / − 45° basis)
DET 1	‘0’ (V/H basis)	‘1’ (V/H basis)

**Table 3:** Detection values of type III DTBS BB84 scheme

	1st DTB	2nd DTB	3rd DTB	4th DTB
DET 0	‘0’ (V/H basis)	‘1’ (V/H basis)	‘0’ (+/− 45° basis)	‘1’ (+/− 45° basis)

**Table 4:** Security concerns in conventional and DTBS schemes of QKD system

	Conventional scheme (BB84 and B92)	DTBS scheme (B92)	DTBS scheme (BB84 Type I)	DTBS scheme (BB84 Type II)	DTBS scheme (BB84 Type III)
Self-correlation	Yes	No	Yes	No	No
Value imbalance	Yes	No	Yes	No	No
Basis imbalance	Yes	No	No	Yes	No

Yes: susceptible to this security concern; No: not susceptible to this security concern.

**Table 5:** Transmittance of different components and overall detection efficiencies of the 1550 nm pump up-conversion detectors

	PPLN_1_	PPLN_2_
PC and WDM at 1310 nm	70 %	74 %
Input coupling of PPLN at 1550 nm*	52 %	71 %
Input coupling of PPLN at 1310 nm*	44 %	59 %
Output coupling of PPLN at 710 nm*	92 %	77 %
Filter before APD*	75 %	88 %
APD efficiency at 710 nm*	70 %	70 %
Overall efficiency	15 %	20 %

* These parameters are provided by the manufactures. Others are measured.

**Table 6:** Measured Sifted-Key Rate

	Point A	Point B	No Switch
Alice to Bob 1	1.57 Mbps	1.96 Mbps	1.98 Mbps
Alice to Bob 2	1.73 Mbps	2.14 Mbps	2.14 Mbps

**Table 7:** Measured PER, Timing Jitter and QBER

	No Switch	Through Switch
PER at Bob 1	22.1 dB	22.0 dB
PER at Bob 2	22.7 dB	22.9 dB
Timing Jitter at Bob 1 (FWHM)	295 ps	295 ps
Timing Jitter at Bob 2 (FWHM)	298 ps	304 ps
QBER at Bob 1	2.3 %	2.4 %
QBER at Bob 2	2.2 %	2.2 %
